# Head and Neck Cancers in Developing Countries

**DOI:** 10.5041/RMMJ.10143

**Published:** 2014-04-28

**Authors:** Poonam Joshi, Sourav Dutta, Pankaj Chaturvedi, Sudhir Nair

**Affiliations:** Head and Neck Surgery, Tata Memorial Hospital, Mumbai, India

**Keywords:** Developing countries, head and neck cancers, health infrastructure, human papillomavirus, tobacco

## Abstract

Head and neck cancers are the most common cancers in developing countries, especially in Southeast Asia. Head and neck cancers are more common in males compared to females. This is mainly attributed to tobacco, areca nut, alcohol, etc. Oral cancers are most common amongst all head and neck squamous cell cancers (HNSCC). HNSCC in the developing world differ from those in the Western world in terms of age, site of disease, etiology, and molecular biology. Poverty, illiteracy, advanced stage at presentation, lack of access to health care, and poor treatment infrastructure pose a major challenge in management of these cancers. The annual GDP (gross domestic product) spent on health care is very low in developing countries compared to the developed countries. Cancer treatment leads to a significant financial burden on the cancer patients and their families. Several health programs have been implemented to curb this rising burden of disease. The main aims of these health programs are to increase awareness among people regarding tobacco and to improve access to health care facilities, early diagnosis, treatment, and palliative care.

## INTRODUCTION

World-wide, the head and neck cancers form the sixth most common cancer.^1^ Head and neck cancer (HNC) is the most common cancer in developing countries.^2^ It is the most common cancer of males in India and the fifth most common in females.^3^ HNC form 21% of the cancers in males and 11% in females in Pakistan.^4^ In India, the age-adjusted rates among females is the highest.^5^

While head and neck cancers form one of the most common cancers in South and Southeast Asian countries, they form only 1%–4% of all cancers in the Western world.^6^ Oral cancers are predominant forms of head and neck squamous cell cancer (HNSCC) in India, Pakistan, and other Southeast Asian countries; oropharyngeal and tongue cancers are common in the Western world.^4^ These differences in site of disease may be related to the prevalent habits in the respective regions.^5^

## RISK FACTORS

Cigarette-smoking and alcohol consumption are the main reasons for HNSCC in the Western population, whereas the use of smokeless tobacco and areca nut is the most common cause of HNSCC in SoutheastAsia.^7^,^8^ The various forms in which smokeless tobacco is used in developing countries include khaini, mava, paan (betel quid), zarda, snuff, mashiri, etc.^9^

Betel quid chewing is the most common form of tobacco chewing in the Asia-Pacific region. Betel quid consists of areca nut, betel leaf, catechu, and slaked lime.^10^ It has been reported from many countries like India, Pakistan, Bangladesh, Sri Lanka, Thailand, Cambodia, Malaysia, Indonesia, China, Philippines, Taiwan, Vietnam, and migrant populations in Europe, Africa, North America, and Australia.^11^ About 10% of the world’s population chew betel quid regularly.^12^ In one study conducted in Southeast Asia, the lower socio-economic groups had higher risk of developing HNC.^13^

Areca nut alone is a confirmed carcinogen and causally associated with a premalignant condition called oral submucous fibrosis (OSMF) and oral cancer.^14^ It is a chronic, debilitating disease of the aerodigestive tract owing to irreversible fibroelastic changes in the lamina propria which lead to stiffness of the oral mucosa resulting in progressive trismus.^15^ This is uncommon in the Western world due to the rarity of areca nut use. In India alone, 5 million people (0.5% of the population of India) have OSMF. It is considered a public health issue in India, South Africa, and many Southeast Asian countries.^16^

Tobacco consumption in India is growing at a rate of 2%–3% per annum.^17^ Tobacco use is expected to cause 8.4 million deaths by 2020, and 70% of these will be in developing countries. In 2010, about 930,000 deaths were estimated to be attributable to tobacco in India.^18^ The high prevalence of tobacco usage has led to increases in disease burden and high health care costs in developing countries. There is a high incidence of smoking reported amongst youth from Bangladesh, India, and Indonesia.^19^ While the incidence of head and neck cancers is decreasing in Europe and North America, it remains unabated in the developing world.^20^

In India, nearly two-thirds of patients present with advanced stages.^13^,^21^ The mean age of patients at presentation of head and neck cancers is the fifth and early sixth decades in Asian populations compared with the seventh and eighth decades in the North American population.^22^–^26^

## HUMAN PAPILLOMAVIRUS (HPV) PREVALENCE

The overall prevalence of HPV in HNSCC is around 50%,^27^ with the highest prevalence in cancers of the tonsil and base of tongue.^28^ The rise in HPV-related cancers has been mainly attributed to the change in sexual practices in the Western world. These patients are younger, have bulky nodes, predominantly oropharynx involvement, equal gender distribution, and have better survival.^29^–^31^ HPV-16 is the most common type, being present in 30.9% of oropharyngeal carcinomas, 16% of oral cancers, and 16.6% of laryngeal cancers. Prevalence of HPV in oral cancers is similar in Europe (16%) and North America (16.1%), but greater in Asia (33%).^32^

The HPV prevalence in India ranges from 33.6% in the Eastern region to 67% in South India and 15% in Western India.^33^,^34^ The prevalences of HPV-6, HPV-11, HPV-16, and HPV-18 were 13%, 20%, 42%, and 47%, respectively.^33^ HPV-16 was most common, followed by HPV-18 and then cross-infection (16 and 18); 41% of patients had multiple HPV infections.^33^ Lesions of the tongue had the highest rate (9 of 11) of HPV infection. Another study showed a rate of HPV infections of 56.3% in cancers of the mandible, 37.5% in cheek, and 38.6% in maxilla. The study also reported that the advanced stages (III, IV) had higher infection rates as compared to earlier stage.^35^

The vaccination of HPV has not been incorporated in the national immunization program of India. There is no evidence to show that HPV vaccination may prevent HNSCC.

## MOLECULAR BIOLOGY OF HEAD AND NECK CANCERS

Between developed and developing countries, there are not only differences in the age, subsite, and habit but also in the molecular biology. The prevalence of the p53 mutation is common in Europe and USA but rare in India. The recent data show the prevalence to be 81% in the Western world. Multiple genetic abnormalities are common in head and cancers in India and Southeast Asia. These include a preponderance of Ha-ras mutations (35%), loss of heterozygosity of Ha-ras (30%), N-ras amplification (28%), and N-myc amplification (29%). These mutations in ras oncogenes are uncommon in the Western world.^36^

## HIGH INCIDENCE RATES IN INDIA

The leading cause of death in males is cancers of the oral cavity, lungs, esophagus, and stomach. In females, the most common site is uterine cervix, breast, and oral cavity. The Cancer Atlas project by the Indian Council for Medical Research (ICMR) has shown the incidences of various cancers in different parts of India.^37^ Aizawl district in the northeastern state of Mizoram has the world’s highest incidence of cancers, in men, of the lower pharynx (11.5 per 100,000 people) and the tongue (7.6 per 100,000 people). Pondicherry has one of the highest incidences of mouth cancer in the world among males (8.9 per 100,000), and Kohima, the capital city of another northeastern state, Nagaland, has the world’s highest incidence of nasopharyngeal cancers.^38^,^39^

## CHALLENGES IN HEALTH CARE

The number of health care institutes dedicated to cancer care is woefully inadequate when compared with Western countries. There are 27 dedicated cancer hospitals (regional cancer centers), and there are about 300 more general or multispecialty hospitals which give cancer care to the patients.^40^ In the year 2010, India spent only 3.7% of its gross domestic product (GDP) on its health sector, which was even lower than the percentage of GDP spent by the other small South Asian countries like Afghanistan (10.4%), Nepal (5.1%), Bhutan (4.3%), or the Maldives (6.2%).^40^,^41^ In the same year, about 71.8% of all healthcare expenditure in India was paid for privately(16.8% in the United Kingdom^42^), with state and central governments contributing 12% and 6.8%, respectively.^43^ This indicates that, with only 3.7% of GDP being spent on healthcare, the government’s contribution is <1% of GDP.^41^

The infrastructure for cancer management is largely inadequate in India. According to the annual report of the Atomic Energy Regulatory Board of the Government of India, till March 2012, 319 institutions across the country had radiotherapy facilities. There were 484 teletherapy units and 343 brachytherapy units at that point of time. Among the teletherapy units, 237 were telecobalt units, and 232 were accelerators.^44^ In the year 2007, only 231 institutions had radiotherapy facilities, with 378 teletherapy units and 266 brachytherapy units.^45^ So it is obvious that there has been a significant improvement of radiotherapy facilities in the last 5 years, especially in the form of accelerator teletherapy units, of which there were 87 in the year 2007, increasing to 232 in 2012. However, for a population of about 1.2 billion, the requirement of radiotherapy machines is about 1,200, which clearly points out that the present resources are far from sufficient.

A study commissioned by the American Society of Clinical Oncology (ASCO) in 2008 showed that, by 2020, there will be 12,500 oncologists available in the USA, and the ratio of cancer patients to oncologists will be 100:1. It was also projected that even if the supply of oncologists were increased by 14%, the requirement for oncologists would increase by 48%.^46^ This discrepancy in oncologist and patient numbers in a developed country like the US reflects that the ratio is expected to be even worse in developing countries where infrastructure as well as medical facilities are scarce. In Zimbabwe, the nurse to patient ratio in provincial hospitals is 1:522, while in district hospitals the ratio may be as high as 1:3,023. Such ratios greatly affect the health care delivery in developing countries.^47^

India is classified as a lower-middle-income group country by the World Bank.^48^ Ninety percent of the oral cancer patients in rural areas belong to the lower or lower-middle socio-economic class, and 3.6% are below the poverty line based on Pareek’s classification.^49^ Around 75% to 80% of patients with cancers present with late-stage incurable disease and hence increased mortality.^50^ One reason for delayed diagnosis is the lack of easy access to healthcare, with one study demonstrating a delay of 67.5 days and 53.7 days in obtaining a first appointment for rural and urban patients, respectively. Other reasons included poor socio-economic status of the patient, cost of care, and high rate of illiteracy.^51^

Another cross-sectional study on head and neck cancers showed that various beliefs of patients (such as “it is ill-fated to have cancer”, “cancer is a curse”), non-availability of transport, ignoring the trivial ulcers in the mouth and believing them to be self-limiting, and prolonged treatment resulting in family stress are important factors in treatment delay.^52^ In addition to inadequate infrastructure, the unequal distribution of health care workers poses a major challenge. The number of health providers per 10,000 population can range from 23.2 in Chandigarh (capital of Punjab and Haryana in the north) to 2.5 in Meghalaya. The majority of the allopathic doctors are located in the south of the country.^53^ Within each geographic region, a greater concentration of trained physicians is seen in urban areas (60%) compared to rural areas. In particular, 74% of all graduate doctors live in urban areas and provide care to 28% of the nation’s population. Similarly, a disproportionate number of nurses and midwives (four times greater) are located in urban compared to rural areas.^43^

## FUTURE OF HEALTH CARE SYSTEM

The major challenge for the management of head and neck cancers is the lower socio-economic class of the majority of the population in developing countries. Along with this, scarcity of infrastructure and health care workers, illiteracy, lack of awareness among the general population about the side effects of tobacco, and prevalent cultural beliefs lead to delays in seeking treatment and, thus, advanced-stage presentation of disease.

In response to the increase in the number of cancer patients, new cancer control programs have been implemented in developing countries with the goals of achieving prevention, strengthening the available cancer treatment facilities, and early diagnosis and treatment.^54^

The government of India has initiated a national cancer control program to combat the increasing incidence of cancer in India. Oncology units have been developed in 82 institutes in both government medical colleges and government hospitals. There are 246 institutions with radiotherapy facilities across the country. The District Cancer Control Programme has been developed to initiate awareness and early detection activities at the district level. Health education has also been promoted under this program.^39^

The need for the future is to have sufficient numbers of trained oncologists and associated medical workers along with infrastructure and government commitment to tackle head and neck cancer as the major disease burden. Along with this, special emphasis needs to be given to prevention programs by the government, as head and neck cancers are potentially preventable.

## Abbreviations:

GDP: gross domestic product
HNC: head and neck cancer
HNSCC: head and neck squamous cell cancers
HPV: human papilloma virus
OSMF: oral submucous fibrosis.
